# Risk for Hospital Readmission following Bariatric Surgery

**DOI:** 10.1371/journal.pone.0032506

**Published:** 2012-03-07

**Authors:** Robert B. Dorman, Christopher J. Miller, Daniel B. Leslie, Federico J. Serrot, Bridget Slusarek, Henry Buchwald, John E. Connett, Sayeed Ikramuddin

**Affiliations:** 1 Department of Surgery, University of Minnesota, Minneapolis, Minnesota, United States of America; 2 Division of Biostatistics, University of Minnesota, Minneapolis, Minnesota, United States of America; University of Colorado, United States of America

## Abstract

**Background and Objectives:**

Complications resulting in hospital readmission are important concerns for those considering bariatric surgery, yet present understanding of the risk for these events is limited to a small number of patient factors. We sought to identify demographic characteristics, concomitant morbidities, and perioperative factors associated with hospital readmission following bariatric surgery.

**Methods:**

We report on a prospective observational study of 24,662 patients undergoing primary RYGB and 26,002 patients undergoing primary AGB at 249 and 317 Bariatric Surgery Centers of Excellence (BSCOE), respectively, in the United States from January 2007 to August 2009.

Data were collected using standardized assessments of demographic factors and comorbidities, as well as longitudinal records of hospital readmissions, complications, and mortality.

**Results:**

The readmission rate was 5.8% for RYGB and 1.2% for AGB patients 30 days after discharge. The greatest predictors for readmission following RYGB were prolonged length of stay (adjusted odds ratio [OR], 2.3; 95% confidence interval [CI], 2.0–2.7), open surgery (OR, 1.8; CI, 1.4–2.2), and pseudotumor cerebri (OR, 1.6; CI, 1.1–2.4). Prolonged length of stay (OR, 2.3; CI, 1.6–3.3), history of deep venous thrombosis or pulmonary embolism (OR, 2.1; CI, 1.3–3.3), asthma (OR, 1.5; CI, 1.1–2.1), and obstructive sleep apnea (OR, 1.5; CI, 1.1–1.9) were associated with the greatest increases in readmission risk for AGB. The 30-day mortality rate was 0.14% for RYGB and 0.02% for AGB.

**Conclusion:**

Readmission rates are low and mortality is very rare following bariatric surgery, but risk for both is significantly higher after RYGB. Predictors of readmission were disparate for the two procedures. Results do not support excluding patients with certain comorbidities since any reductions in overall readmission rates would be very small on the absolute risk scale. Future research should evaluate the efficacy of post-surgical managed care plans for patients at higher risk for readmission and adverse events.

## Introduction

The astonishing rise in obesity prevalence and the marked decline in perioperative mortality over the previous two decades have both contributed to the growing popularity of bariatric surgery. In the period from 1998 to 2003, the number of bariatric procedures performed increased 10-fold [1], and in 2009 alone, 220,000 bariatric surgeries were performed in the United States and Canada [2]. Despite the clinical benefits, the potential for serious and costly major adverse events deters many patients and payers from utilizing its advantages.

Bariatric surgery is safe with a 0.15% to 0.5% 30-day mortality rate [3]–[6], however an appreciable proportion of patients suffer at least one major adverse event within the first 30 days following either Roux-en-Y gastric bypass (RYGB) or adjustable gastric banding (AGB) that results in hospital readmission. A hospital readmission increases the average 180-day cost of a bariatric operation from approximately $27,000 to $65,000 [7]. In response to the high costs of hospital readmission, in 2008 the National Quality Forum indicated that hospital readmission rates would be a central factor in evaluating hospital performance with penalties being levied against hospitals with high readmission rates.

Identifying patient and surgical factors that increase perioperative risk of readmission would improve both the tenability of bariatric surgery for patients and the cost-effectiveness for payers. Comprehensive assessment of patient risks a priori would provide physicians with a framework for either tailoring the selection of intervention or identifying patients most in need of enhanced education or monitoring post-operatively, which could, in turn, reduce the frequency of readmission following bariatric surgery. We have utilized the Bariatric Outcomes Longitudinal Database (BOLD), the largest prospective database of bariatric patient outcomes worldwide, to identify predictors of serious postoperative complications requiring hospital readmission within 30 days of discharge.

## Methods

### Design Overview

We obtained patient data from BOLD collected between January 1, 2007 and August 31, 2009 at 450 Bariatric Surgery Centers of Excellence (BSCOE). Data collection for BOLD was overseen by the Surgical Review Corporation (SRC). Written informed consent was obtained from all patients, and the protocols of this study were approved by the University of Minnesota institutional review board.

The American Society for Metabolic and Bariatric Surgery (ASMBS) founded SRC in 2003 as an independent, nonprofit research organization to oversee compliance and collect data on patient outcomes for accredited BSCOE hospitals. The criteria set forth by the ASMBS for BSCOE certification include: performing at least 125 bariatric surgeries per year in hospitals or 100 bariatric surgeries per year in surgery centers; surgeons must have completed 125 bariatric surgeries during their career and must continue to perform at least 50 bariatric surgeries per year; a multidisciplinary staff including a team of nurses, surgeons, dieticians and other consultants; onsite inspections of BSCOE must be performed every three years; and, hospitals must report their outcomes to the BOLD database.

BOLD, an internet-based database implemented in 2007, represents the largest repository of clinical bariatric patient surgery information in the world with 521 contributing BSCOE. BOLD collects standardized assessments of patient demographics, preoperative morbidities, medication use, surgical procedures, and post-surgical follow-up visits. Additional details regarding the operational procedures of BOLD have been reported previously [8]. Standard protocol for BSCOE requests that patients return to their operating physician for periodic follow-up visits including at least one visit one month post-operatively. At each follow-up visit, visit-specific information is recorded into BOLD on weight, complications, and readmissions since the previous follow-up visit regardless of whether the readmitting hospital was BSCOE-certified.

### Setting and Participants

Enrollment and data entry for BOLD is ongoing, though we excluded surgeries which took place after August 31, 2009 to ensure that all patients had adequate opportunity to follow-up with their surgical center and BSCOE had sufficient time to submit patient data as of the most recent database closing on March 1, 2010. For each patient in the study, we extracted complete data on demographic information, comorbidities, inpatient data, and post-surgical follow-up, including data on readmissions, complications, and mortality. Self-reported data on demographics and comorbid conditions were complete for nearly all patients and suspect data entry errors were rare. Suspect BMI values were replaced with data from an alternate visit. Patients were eligible for inclusion if they underwent a non-revision RYGB or AGB and had complete follow-up through at least 30 days following discharge.

The 30-day follow-up rate among BOLD participants was 91.6% during the study period, however follow-up rates were variable across BSCOE. Analysis of the within-BSCOE relationship between the follow-up rate and readmission rate indicated that centers with lower follow-up rates tended to report lower readmission rates, suggesting that centers with lower follow-up rates underreported their true readmission rates. To ensure high accuracy of outcomes reporting, we excluded BSCOE reporting complete 30-day outcome data for fewer than 90% of their surgical patients. Statistically significant differences between patients from included and excluded BSCOE were observed for several baseline characteristics due to the large sample sizes and correspondingly small standard errors, but these differences were small in magnitude and not clinically relevant. Sensitivity analyses on the effect of restricting participants to highly compliant BSCOE were performed on the readmission and mortality rates under a wide variety of assumptions, but had little effect on the rates due to the relative rarity of the outcome.

### Outcomes and Follow-up

Predictors of interest were demographic, health, and surgical variables. Clinical definitions of comorbid conditions are presented in Table 1. Prolonged length of stay was defined as a hospital stay ≥4 days for laparoscopic RYGB, ≥6 days for open RYGB and ≥2 days for AGB. Our outcome was all-cause hospital readmission within 30 days of discharge requiring hospitalization for >23 hours. We considered patients to be at risk for readmission on the day of discharge. No serious intraoperative complications requiring additional hospitalization were classified as readmissions. Patients were considered at risk for mortality on the day of surgery. Patients who were readmitted and died in the hospital within 30 days of surgery were classified as both readmissions and mortalities; patients who died outside a hospital were classified as mortalities but not readmissions.

**Table 1:** Clinical definitions of pre-existing conditions.

Condition	Clinical Definition
Abdominal hernia	Any history of symptomatic or asymptomatic abdominal hernia
Abdominal/skin pannus	Any current symptoms, including intertriginous irritation, interfering with ambulation, recurrent cellulitis, or ulceration
Alcohol use	Any current alcohol use
Angina	Any chest pain symptoms or angina regardless of exertion
Asthma	Any symptoms of asthma regardless of medication usage
Back pain	Has degenerative changes or positive objective findings, symptoms require narcotic treatment
Bipolar disorder	Confirmed diagnosis of bipolar disorder
Cholelithiasis	Has had gallstones with severe symptoms or has had a cholecystectomy
Congestive heart failure	Any history or symptoms of congestive heart failure (Class I, II, III, and IV)
Depression	At least moderate depression with significant impairment, undergoing medical or therapeutic treatment
DVT/PE	Any history of resolved or recurrent deep venous thrombosis or pulmonary embolism
Fibromyalgia	Any degree of fibromyalgia
Gastroesophageal reflux disease	Symptoms require the use of medical treatment (at least H2 blockers or low-dose proton pump inhibitor)
Gout/hyperuricemia	Has at least symptomatic or asymptomatic hyperuricemia
Hypertension	Requires medical treatment with multiple medications
Ischemic heart disease	Has at least abnormal electrocardiogram, regardless of active ischemia; may include history of myocardial infarction
Lipids	Heightened cholesterol requiring at least single medication
Liver disease	Any history of liver disease, including hepatomegaly or non-normal liver function test
Lower extremity edema	Has symptoms requiring treatment, diuretics, elevation, or hose
Musculoskeletal disease	Has pain with household ambulation, requires surgical intervention, or past joint replacement
Obesity hypoventilation syndrome	Any symptoms including hypoxemia or hypercarbia on room air
Obstructive sleep apnea	Sleep apnea requiring oral appliance, significant hypoxia, or oxygen-dependent
Panic disorder	Confirmed diagnosis of panic disorder
Peripheral vascular disease	Any symptoms of peripheral vascular disease
Personality disorder	Confirmed diagnosis of personality disorder
Psychosocial impairment	Any indicated psychosocial impairment, regardless of ability to perform primary tasks
Pseudotumor cerebri	Any symptoms of pseudotumor cerebri (at least headaches with dizziness, nausea, or pain behind the eyes) with or without visual symptoms
Psychosis	Confirmed diagnosis of psychosis
Pulmonary hypertension	Any symptoms associated with pulmonary hypertension (shortness of breath, dizziness, fainting)
Substance abuse	Any recent substance abuse
Stress urinary incontinence	Frequent stress urinary incontinence, regardless of severity
Tobacco use	Any recent tobacco use
Type-2 diabetes	Diabetes requiring insulin

Abbreviations: DVT, deep venous thrombosis; PE, pulmonary embolism.

### Statistical Analysis

The demographic characteristics and medical histories of patients undergoing the two surgeries were compared with *t*-tests for continuous variables and chi-square tests for categorical variables, and Fisher's exact tests for rare counts. Risk factors for readmission were evaluated using a series of generalized linear mixed-effects models. Demographic, health, and surgical covariates were estimated as fixed effects and a random effect was estimated for BSCOE to account for variation between and correlation within BSCOE in their readmission rates. Those covariates significantly associated with readmission in univariate analysis at a significance level of 0.10 or greater were entered into a multivariate model with an iterative backward selection procedure that continued until all variables were significant at the 0.10 level.

We used expanded mixed-effects logistic models with interactions to examine mediation of risk factors by surgical approach. Readmission risk was higher among open surgeries, however the risk factors for open and laparoscopic RYGB approaches were not significantly different in the expanded models, so we controlled for the higher rate of readmission among open procedures with an additional fixed effect and did not stratify models by surgical approach. All *P* values are two-sided and are unadjusted for multiple comparisons. Statistical analyses were conducted in SAS version 9.2 (SAS Institute Inc., Cary, North Carolina) and graphs were generated in R version 2.14.0 (R Development Core Team, 2011).

## Results

### Patients

**Figure 1:**
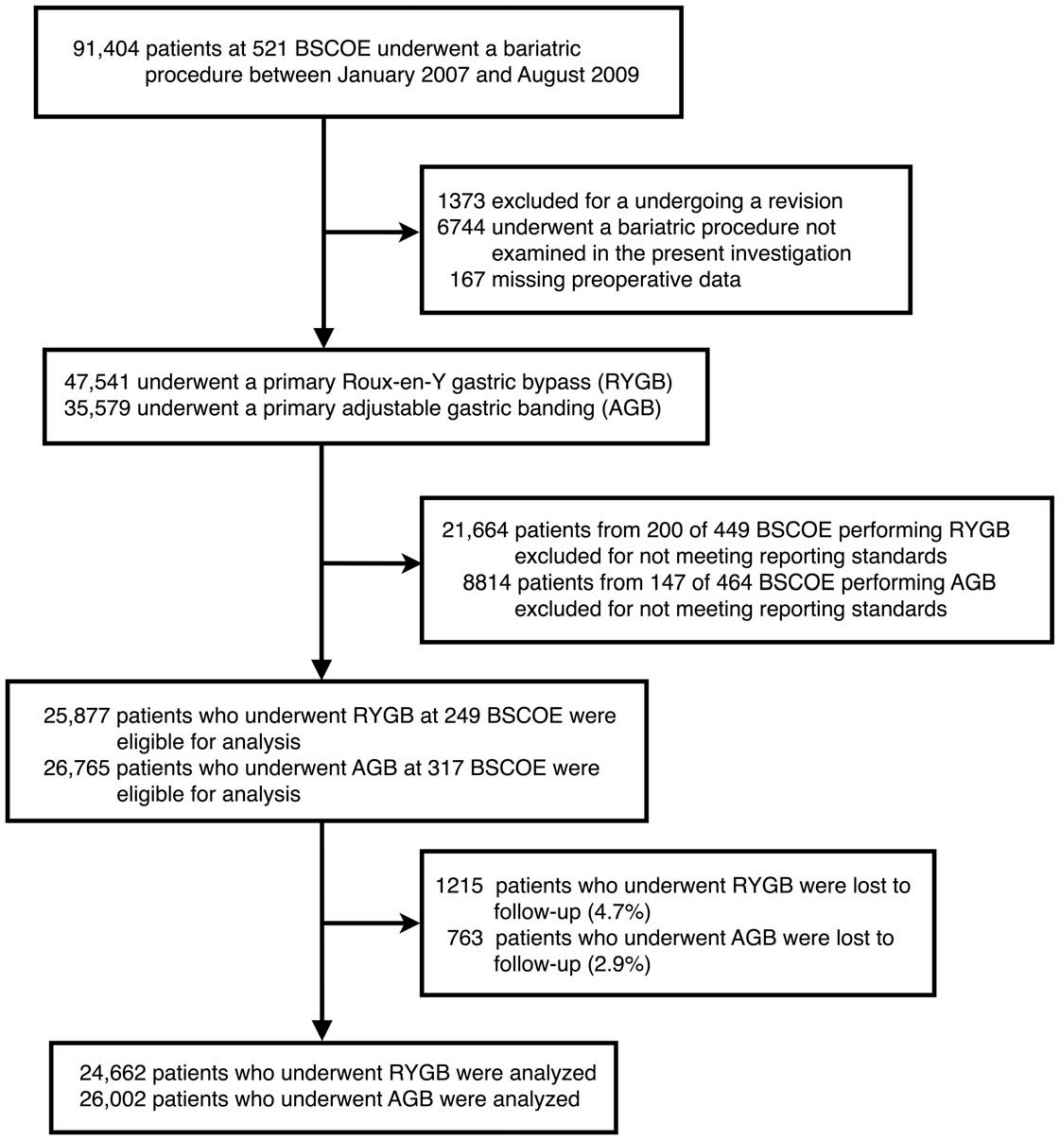
Flow diagram of patient selection.

Figure 1 details the patient selection process. Among BSCOE eligible for analysis, 24,662 RYGB patients from 249 BSCOE and 26,002 AGB patients from 317 BSCOE were followed-up at 30 days. Bariatric surgery patients had a mean age of 45.9±11.9 years and were predominantly female (78.9%) and Caucasian (80.1%). Patients undergoing RYGB had higher BMI and higher prevalence of comorbidities than patients who underwent AGB (Table 2). The laparoscopic approach was employed for 90.7% of RYGB and 99.7% of AGB operations. Prior to discharge, 1,728 (7.7%) laparoscopic RYGB patients, 159 (7.0%) open RYGB patients, and 1,183 AGB patients (4.6%) had a prolonged length of stay.

**Table 2:** Characteristics of Study Participants by Surgery.

	Roux-en-Y Gastric Bypass	Adjustable Gastric Banding	
	(n = 24,662)	(n = 26,002)	
Variable	Mean (SD)/No. (%)	Mean (SD)/No. (%)	*P* value^a^
Demographics			
Age	45.7 (11.6)	46.1 (12.1)	<0.001
Female sex	19,259 (78.1)	20,736 (79.8)	<0.001
Black race	2265 (9.2)	2890 (11.1)	<0.001
Caucasian race	19,973 (81.0)	20,590 (79.2)	<0.001
Disabled	2140 (8.7)	1192 (4.6)	<0.001
Private insurance	20,878 (84.7)	21,478 (82.6)	<0.001
Medicare/Medicaid	2919 (11.8)	2148 (8.6)	<0.001
Medical history			
BMI^b^	47.2 (8.3)	44.2 (6.7)	<0.001
Number of medications	3.8 (4.0)	2.8 (3.5)	<0.001
Comorbidities			
Abdominal hernia	1379 (5.6)	1032 (4.0)	<0.001
Abdominal/skin pannus	2097 (8.5)	1081 (4.2)	<0.001
Alcohol use	7469 (30.3)	7194 (27.7)	<0.001
Angina	914 (3.7)	576 (2.2)	<0.001
Asthma	3312 (13.4)	2707 (10.4)	<0.001
Back pain	2629 (10.7)	1926 (7.4)	<0.001
Bipolar disorder	490 (2.0)	350 (1.4)	<0.001
Cholelithiasis	4614 (18.7)	3843 (14.8)	<0.001
Congestive heart failure	703 (2.9)	435 (1.7)	<0.001
Depression	3589 (14.6)	3065 (11.8)	<0.001
DVT/PE	937 (3.8)	850 (3.3)	0.001
Fibromyalgia	908 (3.7)	694 (2.7)	<0.001
GERD	6473 (26.3)	5629 (21.7)	<0.001
Gout/hyperuricemia	1006 (4.1)	644 (2.5)	<0.001
Hypertension	5850 (23.7)	5051 (19.4)	<0.001
Ischemic heart disease	1299 (5.3)	1152 (4.4)	<0.001
Lipids	6750 (27.4)	6015 (23.1)	<0.001
Liver disease	2160 (8.8)	1102 (4.2)	<0.001
Lower extremity edema	3138 (12.7)	2342 (9.0)	<0.001
Musculoskeletal disease	2637 (10.7)	2137 (8.2)	<0.001
Obesity hypoventilation syndrome	578 (2.3)	506 (2.0)	0.002
Obstructive sleep apnea	7424 (30.1)	5811 (22.4)	<0.001
Panic disorder	2073 (8.4)	1520 (5.9)	<0.001
Peripheral vascular disease	327 (1.3)	227 (0.9)	<0.001
Personality disorder	189 (0.8)	59 (0.2)	<0.001
Psychosocial impairment	4321 (17.5)	3040 (11.7)	<0.001
Pseudotumor cerebri	446 (1.8)	278 (1.1)	<0.001
Psychosis	23 (0.1)	15 (0.1)	0.15
Pulmonary hypertension	1430 (5.8)	1051 (4.0)	<0.001
Substance abuse	105 (0.4)	71 (0.3)	0.004
Stress urinary incontinence	2859 (11.6)	2692 (10.4)	<0.001
Tobacco use	1805 (7.3)	1748 (6.7)	0.009
Type-2 diabetes requiring insulin	3029 (12.3)	2030 (7.8)	<0.001

Abbreviations: BSCOE, bariatric surgery center of excellence; DVT, deep venous thrombosis; GERD, gastroesophageal reflux disease; PE, pulmonary embolism.

a *P* values calculated using *t*-tests for continuous variables and Pearson's chi-squared tests or Fisher's exact tests for categorical variables.

b Body mass index (BMI) was calculated as weight in kilograms divided by height in meters squared.

### Readmission and mortality

**Figure 2:**
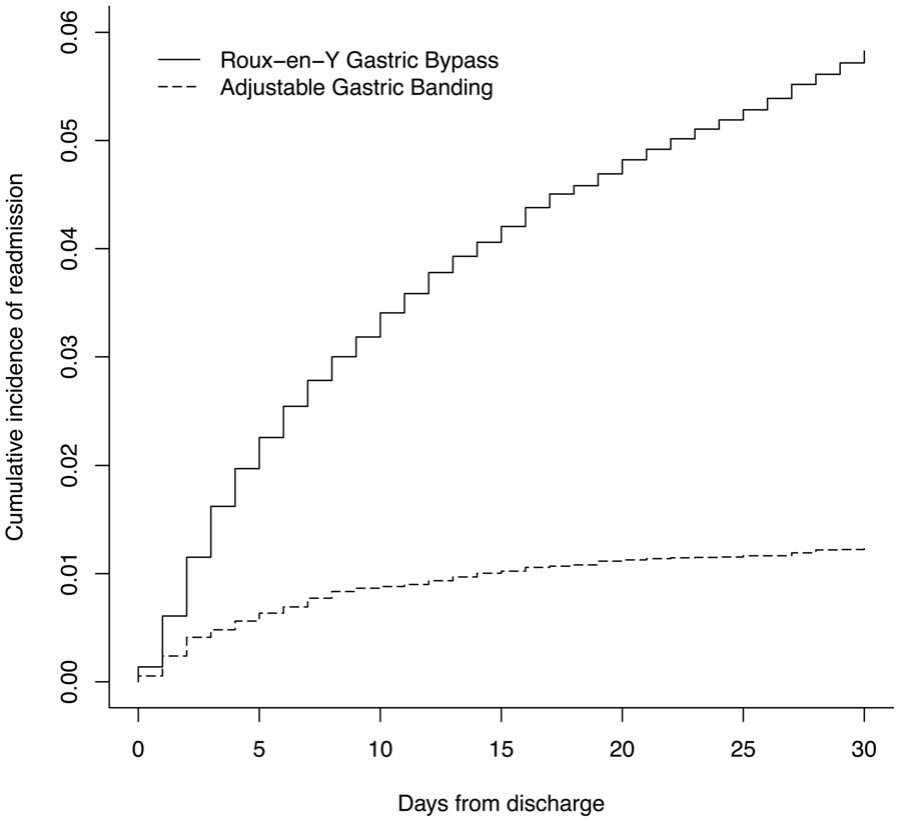
Cumulative incidence of 30-day readmission by surgery.

In the first 30 days after discharge, 1437 (5.8%) RYGB patients and 322 (1.2%) AGB patients were readmitted (Figure 2; *P*<0.001 for difference). Patients undergoing RYGB procedures with the laparoscopic approach had fewer readmissions than patients who underwent RYGB with an open approach (5.6% v. 7.9%, *P*<0.001). The most commonly reported complications at readmission were nausea/vomiting and dehydration for both procedures, though more than one reason could be recorded for a readmission (Table 3). For RYGB, other common complications at readmission were gastrointestinal bleeding, stricture, and obstruction; pneumonia, device-related infection, and obstruction were common complications reported at AGB readmissions.

Within the 30 days of the primary operation, 35 (0.14%) RYGB patients and 6 (0.02%) AGB patients died (*P*<0.001 for difference). For RYGB patients, causes of mortality were sepsis (n = 11), cardiac failure (n = 6), myocardial infarction (n = 4) respiratory failure (n = 4), stroke (n = 2), pulmonary embolus (n = 1), or could not be determined (n = 6). For AGB patients, causes of death were myocardial infarction (n = 3) or indeterminate (n = 3).

**Table 3:** Common Complications at Readmission by Surgery.

	Roux-en-Y Gastric Bypass		Adjustable Gastric Banding	
Most common complications reported at readmission	Complication	No. (%)	Complication	No. (%)
	Nausea/vomiting	346 (24.1)	Nausea/vomiting	57 (17.7)
	Dehydration	170 (11.8)	Dehydration	37 (11.5)
	Gastrointestinal bleeding	82 (5.7)	Device-related infection	27 (8.4)
	Stricture	79 (5.5)	Device-related obstruction	18 (5.6)
	(Internal) obstruction	76 (5.3)	Pneumonia	13 (4.0)
	Anastomotic leakage	58 (4.0)	Wound complication	13 (4.0)
	Wound complications	52 (3.6)	Device-related intolerance	12 (3.7)
	Intra-abdominal abscess	52 (3.6)	Deep venous thrombosis	11 (3.4)
30-day readmission rate		1437 (5.8)		322 (1.2)
30-day mortality rate		35 (0.14)		6 (0.02)

BSCOE could report more than one complication at readmission, so the reported percentages reflect the proportion of all readmissions involving those complications. This table lists the eight most-reported complications per surgery and does not add to 100%. A device-related obstruction was a complication of the device causing intestinal obstruction, whereas device-related intolerance is an unspecified complication due the device, implant, and graft.

### Predictors of readmission

Table 4 presents univariate and multivariate risk factor analyses for readmission. After adjusting for other significant covariates, prolonged length of stay more than doubled the odds of readmission for a RYGB patient and the open surgical approach nearly doubled odds of readmission. Patients with current symptoms of clinical depression or psychosocial impairment, peripheral vascular disease, pseudotumor cerebri, or those with a previous history of gallstones or cholecystecomy were more likely to be readmitted than those without those symptoms. The number of medications used preoperatively was also associated with higher readmission rates, and an African-American patient had 34% higher odds of readmission compared to a Caucasian individual holding other factors constant.

**Table 4:** Univariate and Multivariate Models of Risk Factors for 30-Day All-Cause Hospital Readmission by Surgery.

	Roux-en-Y Gastric Bypass		Adjustable Gastric Banding	
	Univariate models	Multivariate model	Univariate models	Multivariate model
Variable	OR (95% CI)	OR (95% CI)	OR (95% CI)	OR (95% CI)
Age (5 years)	1.00 (0.98–1.03)	-	1.11 (1.06–1.17)	-
Male gender	0.94 (0.83–1.08)	-	1.68 (1.32–2.14)	1.45 (1.12–1.87)
Race (ref. Caucasian)				
Black/African American	1.32 (1.10–1.58)	1.34 (1.11–1.62)	0.63 (0.41–0.97)	-
Hispanic/Latino	1.01 (0.77–1.31)	1.05 (0.81–1.38)	0.97 (0.55–1.70)	-
Other	1.06 (0.74–1.51)	1.07 (0.74–1.53)	0.83 (0.44–1.55)	-
ASA classification^a^ (ref. “1”)				
2/3 – mild systemic disease	1.45 (0.81–2.61)	-	1.64 (0.77–3.47)	1.41 (0.66–2.99)
4/5 – severe disease	2.23 (1.20–4.16)	-	4.49 (1.88–10.7)	2.44 (1.01–5.89)
BMI^b^ (ref. 45–49.9)				
30–34.9	1.07 (0.71–1.61)	-	0.81 (0.42–1.57)	-
35–39.9	1.02 (0.86–1.22)	-	1.25 (0.88–1.78)	-
40–44.9	0.98 (0.84–1.15)	-	1.19 (0.84–1.67)	-
50–54.9	1.12 (0.94–1.35)	-	1.48 (0.96–2.29)	-
55–59.9	1.15 (0.91–1.46)	-	1.93 (1.11–3.33)	-
60+	1.35 (1.10–1.66)	-	2.52 (1.47–4.33)	-
Employment status (ref. Employed)				
Disabled	1.55 (1.30–1.85)	-	2.82 (1.95–4.09)	1.79 (1.21–2.65)
Retired	1.18 (0.96–1.45)	-	1.98 (1.43–2.73)	1.43 (1.02–2.01)
Unemployed	1.26 (1.03–1.53)	-	1.15 (0.68–1.93)	1.02 (0.60–1.72)
Payment method (ref. private insurance)				
Self-payer	0.67 (0.46–0.97)	0.69 (0.48–1.01)	1.10 (0.72–1.69)	-
Medicare/Medicaid	1.46 (1.24–1.70)	1.12 (0.95–1.32)	2.22 (1.63–3.03)	-
Number of medications (5 med interval)	1.25 (1.17–1.34)	1.10 (1.02–1.19)	1.50 (1.30–1.72)	-
Open surgical method	1.93 (1.56–2.39)	1.78 (1.44–2.20)	2.48 (0.59–10.37)	-
Prolonged length of stay	2.47 (2.11–2.89)	2.28 (1.95–2.68)	3.08 (2.19–4.33)	2.32 (1.63–3.30)
Comorbidities				
Alcohol use	0.76 (0.66–0.87)	0.82 (0.71–0.94)	0.83 (0.63–1.08)	-
Angina	1.41 (1.09–1.83)	-	2.35 (1.43–3.85)	1.58 (0.95–2.63)
Asthma	1.26 (1.09–1.46)	-	1.77 (1.32–2.37)	1.52 (1.12–2.05)
Cholelithiasis	1.29 (1.13–1.48)	1.18 (1.03–1.36)	1.25 (0.93–1.68)	-
Depression	1.34 (1.15–1.56)	1.18 (1.01–1.39)	1.18 (0.85–1.65)	-
DVT/PE	1.49 (1.17–1.91)	1.24 (0.96–1.60)	2.79 (1.78–4.37)	2.09 (1.32–3.29)
GERD	1.22 (1.08–1.38)	-	1.54 (1.20–1.97)	1.30 (1.01–1.68)
Ischemic heart disease	1.49 (1.21–1.83)	1.21 (0.97–1.51)	1.86 (1.24–2.79)	-
Lower extremity edema	1.24 (1.06–1.45)	-	1.98 (1.45–1.90)	1.36 (0.99–1.89)
Obstructive sleep apnea	1.10 (0.98–1.24)	-	1.97 (1.56–2.49)	1.45 (1.13–1.87)
Psychosocial impairment	1.36 (1.17–1.57)	1.19 (1.02–1.39)	0.97 (0.69–1.38)	-
Pseudotumor cerebri	1.75 (1.20–2.55)	1.63 (1.11–2.39)	1.82 (0.79–4.22)	-
Peripheral vascular disease	1.78 (1.23–2.57)	1.44 (0.99–2.11)	1.28 (0.47–3.50)	-

Abbreviations: ASA, American Society of Anesthesiologists; CI, confidence interval; DVT, deep venous thrombosis; GERD, gastroesophageal reflux disease; OR, odds ratio; PE, pulmonary embolism. Ref denotes the reference group of a categorical variable. The variables for participation of surgical resident, COE volume, abdominal hernia, abdominal pannus, back pain, bipolar disorder, congestive heart failure, fibromyalgia, gout/hyperuricemia, hypertension, lipids, liver disease, musculoskeletal disease, obesity hypoventilation syndrome, panic disorder, personality disorder, psychosis, pulmonary hypertension, substance abuse, stress urinary incontinence, and type-2 diabetes were not significant at *P*≤.10 in univariate analysis and/or not significant in either multivariate model for either surgery and are not shown. Dashes indicate that the variable was not included in the multivariate model because it was removed either for not meeting the significance threshold in the univariate model or for being removed in the backwards selection procedure.

For AGB, prolonged length of stay also doubled a patient's odds of readmission. Male patients had nearly 50% greater odds of readmission than female patients, and disabled and retired employment statuses were more likely to have been readmitted than employed individuals after controlling for other significant demographic and health factors. Patients undergoing AGB with symptomatic asthma, gastroesophageal reflux disease (GERD), obstructive sleep apnea (OSA), or a history of deep venous thrombosis or pulmonary embolism (DVT/PE) had significantly higher odds of being readmitted within 30 days compared to patients without those medical complications.

## Discussion

The overall reduction in mortality and the resolution of chronic diseases such as type 2 diabetes are substantial following bariatric surgery [9]–[15]. However, the potential for serious complications is a barrier for patients and payers to utilizing the long-term advantages offered by bariatric surgery. The penalizing of hospitals for early readmissions is already underway, and several states are imposing mandates that call for further reductions in readmissions. Primary care providers and surgeons alike will see substantial decreases in reimbursements for readmitted patients, and it is therefore imperative that systems be in place to prevent the occurrence of readmissions. In this study, we have identified factors predictive of severe events requiring hospital readmission within 30 days of RYGB or AGB in the largest prospective bariatric cohort to date and have established that short-term risk for readmission is low for both procedures and risk profiles are largely unique to each procedure.

We observed a RYGB readmission rate nearly five times higher than AGB. Previously reported hospital readmission rates for bariatric surgery vary widely in the literature [6], [7], [16]–[20]. Possible explanations for these disparities may be due to differences in the patient populations, the definition of a hospital readmission, the proportion of patients within a sample undergoing open versus laparoscopic procedures, or surgeon experience.

The higher readmission and mortality rates for RYGB relative to AGB might suggest that AGB is preferable, however such risks must be weighed with the treatment outcomes. RYGB has been shown to result in greater weight loss and superior improvement in comorbid illness [11], [21]–[23]. Our group recently reported on greater one-year improvements among patients with type 2 diabetes with respect to weight loss, hemoglobin A_1_C, medication scores, and rates of diabetes resolution for RYGB patients compared to matched AGB controls [11]. Short-term complications must be weighed against the long-term benefits and complications of each procedure.

The clearest predictors of readmission following RYGB were the use of the open surgical approach and prolonged length of stay. While open procedures are justified for certain complex cases [24], our results, when considered with previous research [25], suggest that laparoscopic techniques should be preferred to open surgery in the absence of contraindications for laparoscopy. Patients with previous histories of bariatric surgery or other anatomical abnormalities may be best suited for open surgeries and would understandably be at higher risk for readmission. However, 7.6% of hospitals (19 of 249 BSCOE) conducted >80% of their RYGB procedures using the open approach and accounted for 46.6% of all open procedures in the database, suggesting that the open approach may be often dictated by surgeon preference in these hospitals rather than case difficulty.

The influence of serious comorbid disease on readmission risk for RYGB patients is expected, though the causal pathway of elevated risk for African-Americans is less clear. We suspect that the association with race may have been confounded by unmeasured variables such as surgical preparation, social support, economic status, or dietary intake. For AGB, readmission risk factors were quite different from those identified with RYGB with the exception of prolonged length of stay and severe ASA score: disability status, asthma, male gender, history of DVT/PE, and the presence of OSA or GERD.

Surprisingly, the profiles of risk factors for readmission were almost entirely distinct for the AGB and RYGB procedures. Prolonged length of stay following surgery was one of the only factors that significantly predicted readmissions in both surgical populations in multivariate analysis. That procedure-specific risk factors contrast so greatly between the two procedures is an important finding potentially overlooked by prior investigations. Previous studies have chosen to pool patients across procedures for analysis assuming that the underlying risk factors were the same [5], [26]. While several of our results are complementary, the choice to aggregate surgical patients may account for some important differences in results. Some previous studies examining readmission rates have identified high BMI as a risk factor for readmission [5], [17], while our own did not. Risk analyses that pooled patients from multiple procedures may have observed an artificial inflation of risk for high-BMI patients who tend to undergo RYGB, which has a significantly higher readmission rate than AGB. The relationship between BMI and readmission risk may also have been confounded by a less complete comorbidity profile in risk models, since many conditions are more prevalent among individuals of greater weight. The ability to examine the role of a very extensive list of comorbidities is a major strength of this analysis.

Many of the identified risk factors, while complex, multifactorial, and often not necessarily modifiable, provide an impetus to follow patients at higher risk for readmission more aggressively following discharge. Prolonged length of stay, for example, was identified as an important risk factor yet the reasons for the longer stay varied widely in BOLD; both preoperative and perioperative factors can interact to influence the duration of a patient's stay. Despite this heterogeneity, prolonged length of stay could be utilized as a prompt for enhanced post-discharge monitoring in patients at higher risk for readmission. Intervention studies are needed to determine if and how enhanced monitoring, adjunctive treatments, or additional education might reduce readmission rates for high-risk patients. Certainly, enhanced monitoring is unlikely to prevent more serious readmissions such as those in patients who develop gastrointestinal leaks or obstructions. Further, it is unknown at this time how much effort would need to be applied to significantly lower the current readmission rates that are already acceptably low. However, it may be possible to impact the most prevalent reasons for readmission, nausea and vomiting, by establishing infusion centers for patients suffering from a slow return of bowel function and dehydration.

It is important to recognize the magnitude of relative risk differences associated with the predictors of readmission in this analysis; primarily, comorbid conditions must be weighed with the absolute risk for each procedure. For example, a relative risk of 1.5 for a high-risk patient group compared to a group of typical patients would equate to an increase of the readmission rate from 5.8% to 8.7% for RYGB and from 1.2% to 1.8% for AGB, or absolute risk differences of 2.9% and 0.6%, respectively. Patients, payers, and practitioners alike may find these higher risks acceptable if outweighed by the benefits of surgery, which are often greatest among patients with more severe comorbidity profiles. For these reasons, we deem that the current results do not support patient selection, but rather highlight patient groups that could benefit from appropriate preventative or educational efforts, and possibly, closer post-discharge follow-up.

This study has several limitations. The exclusion of centers with low follow-up rates is the most important limitation, since it is possible that BSCOE excluded for low follow-up rates may have been the hospitals with the highest readmission rates, as well. Our sensitivity analyses comparing included and excluded centers did not indicate that there were substantive clinical differences between the patient populations (Tables 5 and 6), and if such a bias were present, it is highly unlikely that an underestimation of readmission rates would have a considerable impact on the strength or direction of the risk factors themselves. Long-term follow-up in BOLD was limited and precluded the examination of readmissions occurring beyond 30 days. RYGB patients continue to require readmission up to and beyond one year, and the need for band revisions generally do not occur within the first 30 days of surgery; however, the greatest proportion of readmissions occur within 30 days [17], [18], so the current study likely captures the most important risk factors for readmission.

**Table 5:** Comparison of RYGB Patients from Included and Excluded BSCOE.

	Included BSCOE	Excluded BSCOE	
	n = 25,877	n = 21,664	
Variable	Mean (SD) or No. (%)	Mean (SD) or No. (%)	*P* value^a^
BSCOE	249 (55.5)	200 (44.5)	-
Age	45.6 (11.6)	45.1 (11.5)	<0.001
Female sex	20,162 (77.9)	17,051 (78.7)	0.04
Race			<0.001
Black	2406 (9.3)	2373 (11.0)	
Caucasian	20,909 (80.8)	15,808 (73.0)	
Hispanic/Latino	1789 (6.9)	1799 (8.3)	
Other	773 (3.0)	1684 (7.8)	
ASA Classification			<0.001
1 – normal, healthy	338 (1.3)	1337 (6.2)	
2/3 – mild systemic disease	24,072 (93.0)	19,276 (89.0)	
4/5 – severe/very severe disease	1467 (5.7)	1051 (4.9)	
BMI^b^	47.2 (8.3)	47.3 (8.3)	0.37
Employment status			<0.001
Employed	19,879 (76.8)	16,885 (77.9)	
Disabled	2273 (8.8)	1680 (7.8)	
Retired	1819 (7.0)	1425 (6.6)	
Unemployed	1906 (7.4)	1674 (7.7)	
Payment Information			0.10
Private insurance	21,876 (84.5)	18,467 (85.2)	
Self-payer	903 (3.5)	731 (3.4)	
Medicare/Medicaid	3098 (12.0)	2466 (11.4)	
Number of medications	3.8 (4.0)	3.2 (3.8)	<0.001
Comorbidities			
Abdominal hernia	1456 (5.6)	939 (4.3)	<0.001
Abdominal/skin pannus	2180 (8.4)	1111 (5.1)	<0.001
Alcohol use	7798 (30.1)	6167 (28.5)	<0.001
Angina	976 (3.8)	488 (2.3)	<0.001
Asthma	3477 (13.4)	2903 (13.4)	0.91
Back pain	2754 (10.6)	1982 (9.2)	<0.001
Bipolar disorder	521 (2.0)	446 (2.1)	0.74
Cholelithiasis	4836 (18.7)	3470 (16.0)	<0.001
Congestive heart failure	751 (2.9)	437 (2.0)	<0.001
Depression	3782 (14.6)	2890 (13.3)	<0.001
DVT/PE	971 (3.8)	612 (2.8)	<0.001
Fibromyalgia	949 (3.7)	670 (3.1)	<0.001
GERD	6781 (26.2)	5073 (23.4)	<0.001
Gout/hyperuricemia	1036 (4.0)	655 (3.0)	<0.001
Hypertension	6115 (23.6)	4587 (21.2)	<0.001
Ischemic heart disease	1374 (5.3)	917 (4.2)	<0.001
Lipids	7014 (27.1)	5562 (25.7)	<0.001
Liver disease	2264 (8.8)	1091 (5.0)	<0.001
Lower extremity edema	3287 (12.7)	2370 (10.9)	<0.001
Musculoskeletal disease	2766 (10.7)	2319 (10.7)	0.96
Obesity hypoventilation syndrome	608 (2.4)	525 (2.4)	0.60
Obstructive sleep apnea	7779 (30.1)	6474 (29.9)	0.67
Panic disorder	2175 (8.4)	1483 (6.9)	<0.001
Peripheral vascular disease	247 (1.3)	241 (1.1)	0.02
Personality disorder	196 (0.8)	52 (0.2)	<0.001
Psychosocial impairment	4544 (11.6)	3138 (14.5)	<0.001
Pseudotumor cerebri	467 (1.8)	635 (2.9)	<0.001
Psychosis	23 (0.1)	24 (0.1)	0.47
Pulmonary hypertension	1502 (5.8)	727 (3.4)	<0.001
Substance abuse	111 (0.4)	96 (0.4)	0.83
Stress urinary incontinence	2968 (11.5)	2683 (12.4)	0.002
Tobacco use	1910 (7.4)	1447 (6.7)	0.003
Type-2 diabetes	3172 (12.3)	2493 (11.5)	0.01

Abbreviations: BSCOE, bariatric surgery center of excellence; DVT, deep venous thrombosis; GERD, gastroesophageal reflux disease; PE, pulmonary embolism.

a *P* values calculated using a *t*-test for continuous variables and Pearson's chi-squared test or Fisher's exact test for categorical variables.

b Body mass index (BMI) was calculated as weight in kilograms divided by height in meters squared.

**Table 6:** Comparison of AGB Patients from Included and Excluded BSCOE.

	Included BSCOE	Excluded BSCOE	
	n = 26,765	n = 8814	
Variable	Mean (SD) or No. (%)	Mean (SD) or No. (%)	*P* value^a^
BSCOE	317 (68.3)	147 (31.7)	-
Age	46.1 (12.1)	46.5 (12.3)	0.01
Female sex	21,319 (79.7)	6919 (78.5)	0.02
Race			<0.001
Black/African American	2979 (11.1)	942 (10.7)	
Caucasian	21,171 (79.1)	6183 (70.2)	
Hispanic/Latino	1089 (4.1)	674 (7.7)	
Other	1526 (5.7)	1015 (11.5)	
ASA Classification			0.004
1 – normal, healthy	1388 (5.2)	381 (4.3)	
2/3 – mild systemic disease	24,635 (92.0)	8200 (93.0)	
4/5 – severe/very severe disease	742 (2.8)	233 (2.6)	
BMI^b^	44.2 (6.7)	44.2 (7.2)	0.62
Employment Status			<0.001
Employed	21,937 (82.0)	7132 (80.9)	
Disabled	1240 (4.6)	527 (6.0)	
Retired	2313 (8.6)	783 (8.9)	
Unemployed	1275 (4.8)	372 (4.2)	
Payment Information			<0.001
Private Insurance	22,085 (82.5)	7169 (81.3)	
Self-Payer	2447 (9.1)	652 (7.4)	
Medicare/Medicaid	2233 (8.3)	993 (11.3)	
Number of medications	2.8 (3.5)	3.1 (3.5)	<0.001
Comorbidities			
Abdominal hernia	1054 (3.9)	486 (5.5)	<0.001
Abdominal/Skin pannus	1107 (4.1)	381 (4.3)	0.44
Alcohol use	7407 (27.7)	2750 (31.2)	<0.001
Angina	596 (2.2)	158 (1.8)	0.01
Asthma	2788 (10.4)	893 (10.1)	0.45
Back pain	1999 (7.5)	626 (7.1)	0.25
Bipolar disorder	364 (1.4)	140 (1.6)	0.12
Cholelithiasis	3938 (14.7)	1146 (13.0)	<0.001
Congestive heart failure	445 (1.7)	140 (1.6)	0.66
Depression	3144 (11.8)	767 (8.7)	<0.001
DVT/PE	878 (3.3)	218 (2.5)	<0.001
Fibromyalgia	708 (2.7)	240 (2.7)	0.70
GERD	5783 (21.6)	1563 (17.7)	<0.001
Gout/hyperuricemia	661 (2.5)	414 (4.7)	<0.001
Hypertension	5197 (19.4)	1693 (19.2)	0.67
Ischemic heart disease	1197 (4.5)	359 (4.1)	0.12
Lipids	6192 (23.1)	2278 (25.9)	<0.001
Liver disease	1151 (4.3)	438 (5.0)	0.008
Lower extremity edema	2413 (9.0)	671 (7.6)	<0.001
Musculoskeletal disease	2219 (8.3)	814 (9.1)	0.02
Obesity hypoventilation syndrome	517 (1.9)	150 (1.7)	0.17
Obstructive sleep apnea	5993 (22.4)	1931 (21.9)	0.34
Panic disorder	1567 (5.9)	541 (6.1)	0.33
Peripheral vascular disease	234 (0.9)	89 (1.0)	0.24
Personality disorder	62 (0.2)	10 (0.1)	0.04
Psychosocial impairment	3145 (11.8)	1034 (11.7)	0.96
Pseudotumor cerebri	290 (1.1)	88 (1.0)	0.55
Psychosis	15 (0.1)	6 (0.1)	0.62
Pulmonary hypertension	1084 (4.1)	167 (1.9)	<0.001
Substance abuse	73 (0.3)	35 (0.4)	0.07
Stress urinary incontinence	2748 (10.3)	833 (9.5)	0.03
Tobacco use	1813 (6.8)	568 (6.4)	0.28
Type-2 diabetes	2093 (7.8)	670 (7.6)	0.51

Abbreviations: BSCOE, bariatric surgery center of excellence; DVT, deep venous thrombosis; GERD, gastroesophageal reflux disease; PE, pulmonary embolism.

a *P* values calculated using a *t*-test for continuous variables and Pearson's chi-squared test or Fisher's exact test for categorical variables.

b Body mass index (BMI) was calculated as weight in kilograms divided by height in meters squared.

**Figure 3:**
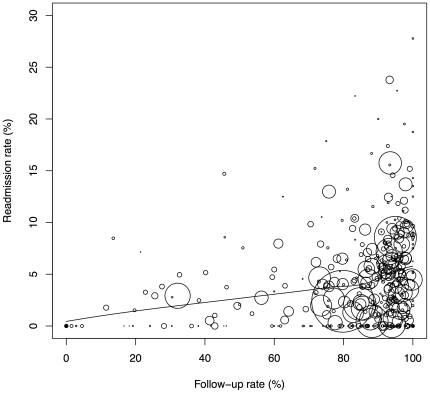
Loess plot of RYGB readmission rates on follow-up rates. Circles represent BSCOE hospitals with the size weighted by the number of patients who underwent the procedure in the hospital during the study period.

**Figure 4:**
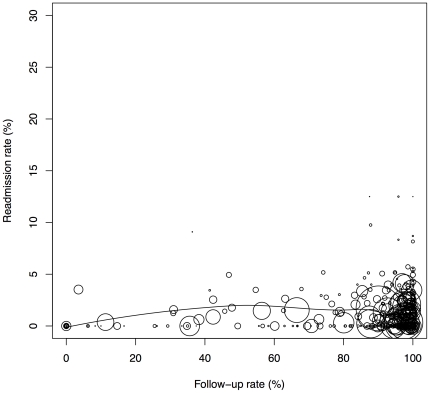
Loess plot of AGB readmission rates on follow-up rates. Circles represent BSCOE hospitals with the size weighted by the number of patients who underwent the procedure in the hospital during the study period.

The present analysis was also unable to control for surgeon volume, an important factor in readmission [16], [19], [20]. however surgeons reporting to BOLD must log over 50 cases annually in order to maintain BSCOE certification. Also, the distribution of readmission rates was not consistent with a uniform rate across centers (Figures 3 and 4). An appreciable number of BSCOE, for AGB in particular, reported readmission rates considerably lower than would be expected under a constant rate across centers of varying surgical volume. We suspect that unusually low BSCOE readmission rates reflect unmeasured variables such as surgical experience, and those with high rates of readmission could be indicative of either surgeon inexperience, case difficulty, or surgeon preference for open procedures. Data entered into BOLD is self-reported by BSCOE, so post-discharge events are potentially underreported in the database, though our selection of centers with high follow-up rates was undertaken to offset potential underreporting in the larger database.

Finally, the observational nature of the study precludes causal inference about risk factors. Given that examination of factors influencing relatively rare events like readmissions requires thousands of patients to be adequately powered to assess differences in risk, it is unlikely that randomized studies of these factors will ever be performed. Therefore, decisions on patient selection and risk calculations will inevitably be based on large prospective observational databases like BOLD. Nested case-control studies, in which more extensive collection of possible explanatory variables is performed, may shed light on the problem of unmeasured confounders in the BOLD dataset.

In conclusion, we have characterized patterns of risk for readmission associated with patient and intraoperative factors for the two most common bariatric procedures in the largest prospective cohort of bariatric surgery patients to date. While the overall readmission rates for both procedures are low, the present results may prove to be an important clinical tool in the development of patient education programs, algorithms for procedure selection, and follow-up plans. In an effort to maximize patient benefit and cost-effectiveness of bariatric surgery and to reduce penalties from payers, primary care providers and surgeons should understand patient-specific risks to optimize clinical care for patients when both selecting for and immediately following their bariatric operation.
